# Supplementary figures and images for: Protective effect and mechanism of Lacticaseibacillus paracasei 207-27 administration on colitis in antibiotic-exposed mice in early life
Source: Microbiol Spectr. 2025 Oct 31;13(12):e02762-24. doi: 10.1128/spectrum.02762-24 (PMC12671169; doi:10.1128/spectrum.02762-24)

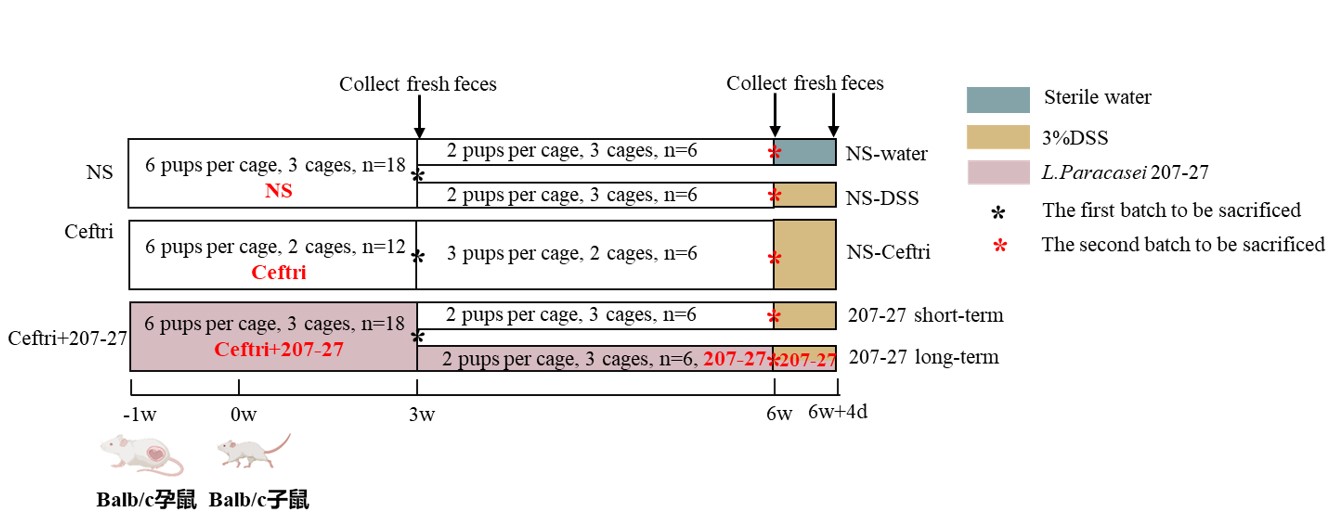

Supplement: Figure S1 — Schematic of grouping, sample size, and intervention. [file spectrum.02762-24-s0001.jpg]

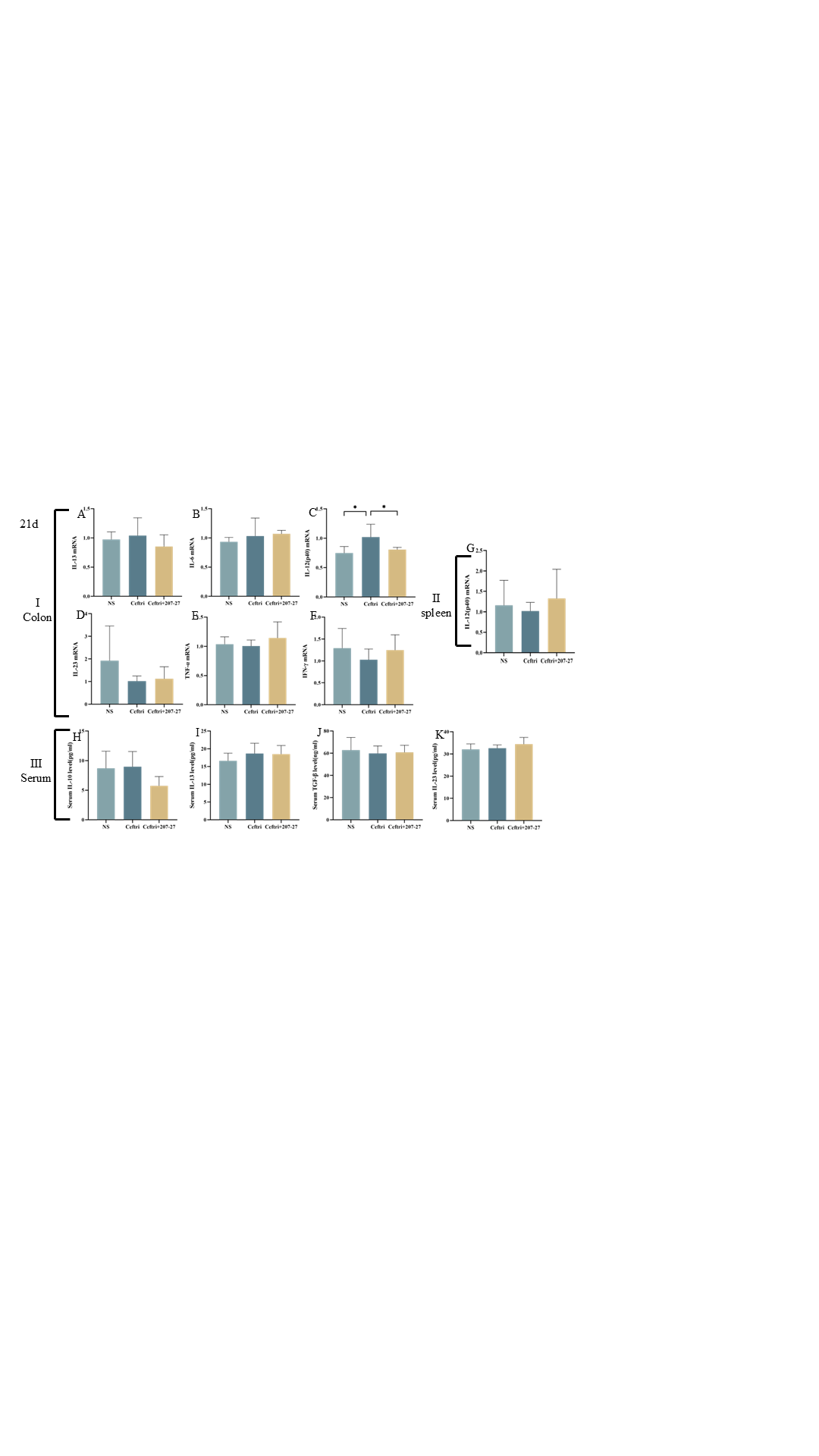

Supplement: Figure S2 — Local and systemic immunities after intervention on day 21 (n = 5–8). [file spectrum.02762-24-s0002.tiff]

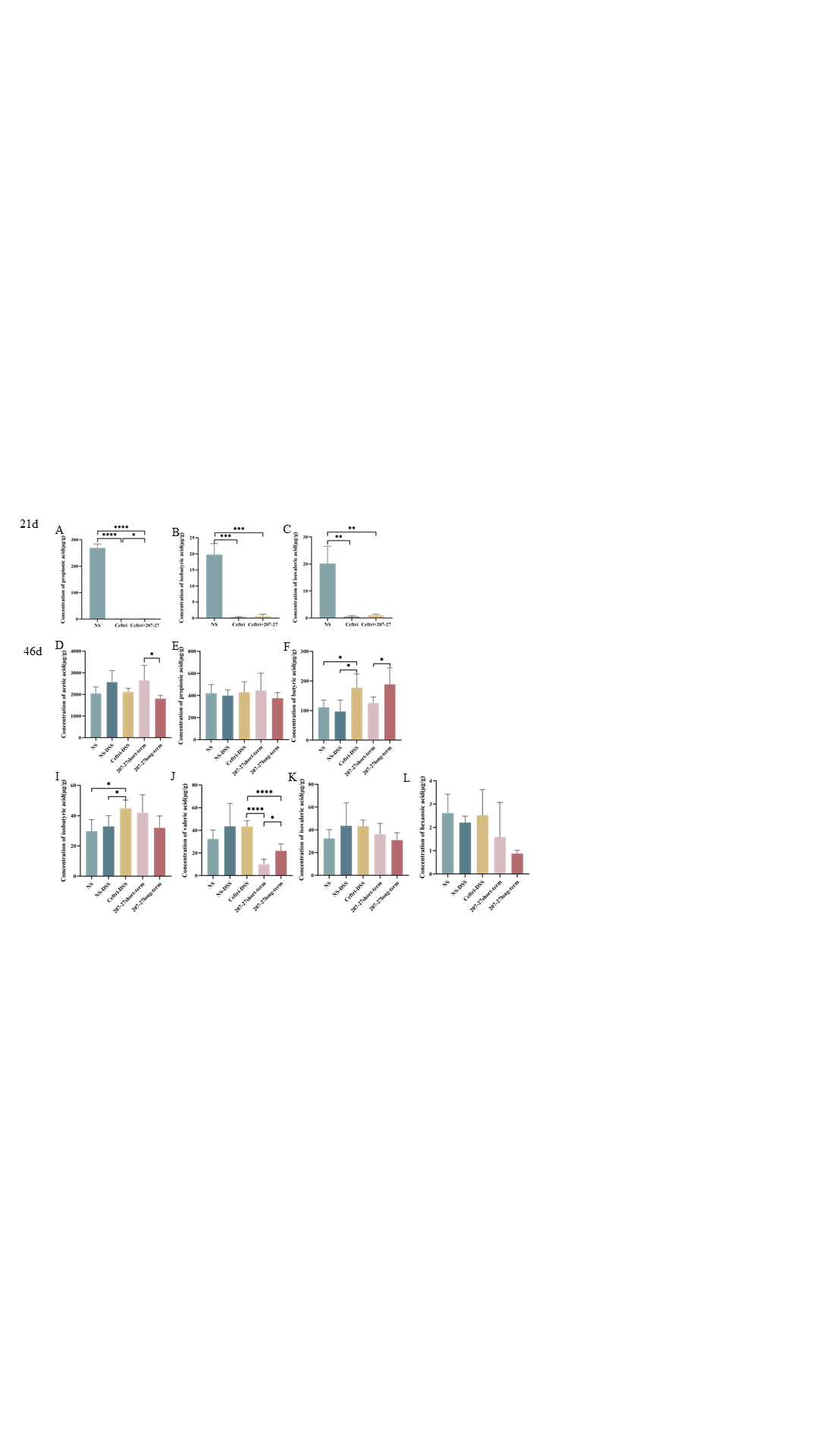

Supplement: Figure S3 — Effects on metabolites on days 21 and 46 (n = 5). [file spectrum.02762-24-s0003.tiff]

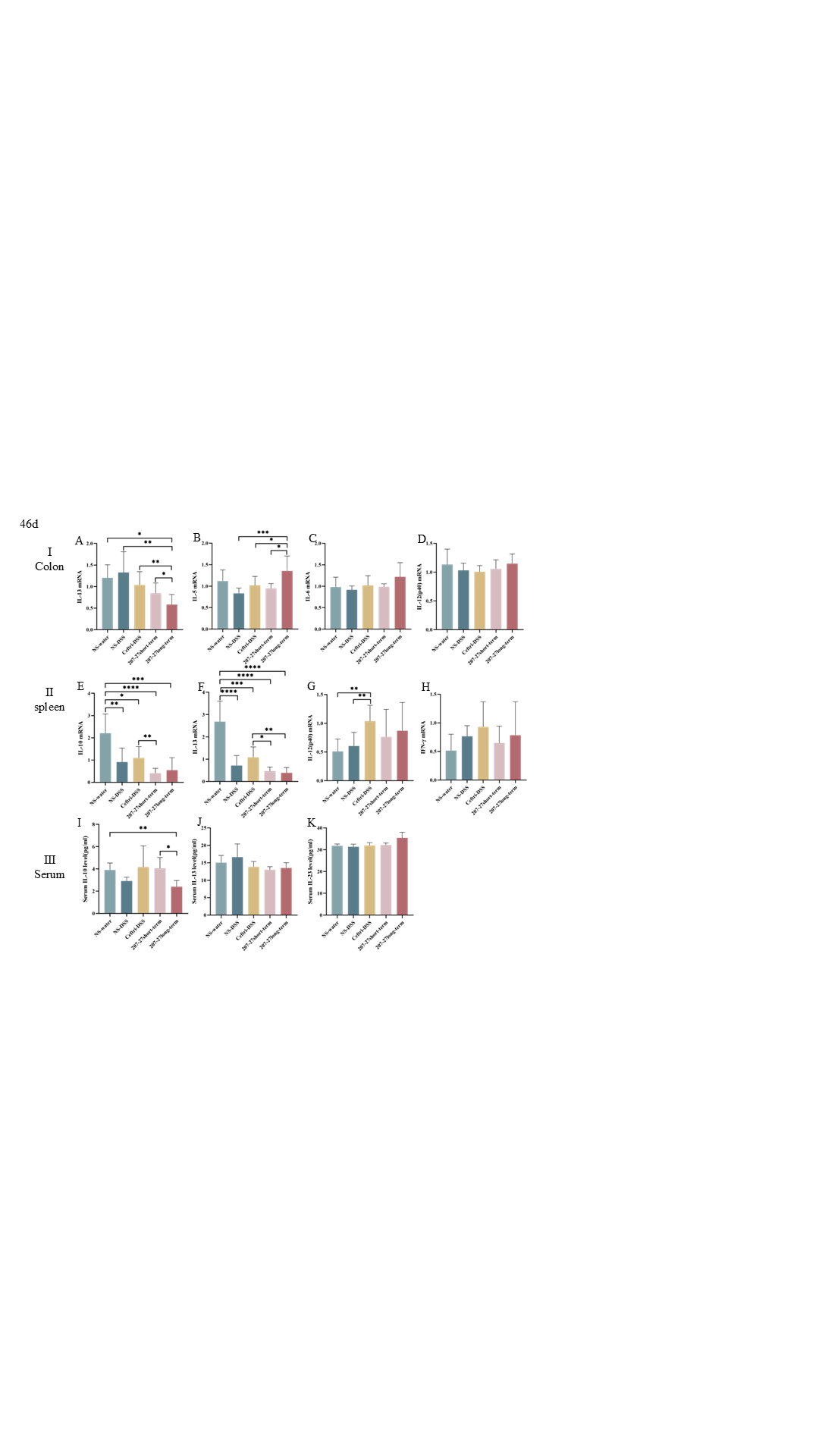

Supplement: Figure S4 — Local and systemic immunities after intervention on day 46 (n = 5–8). [file spectrum.02762-24-s0004.tiff]

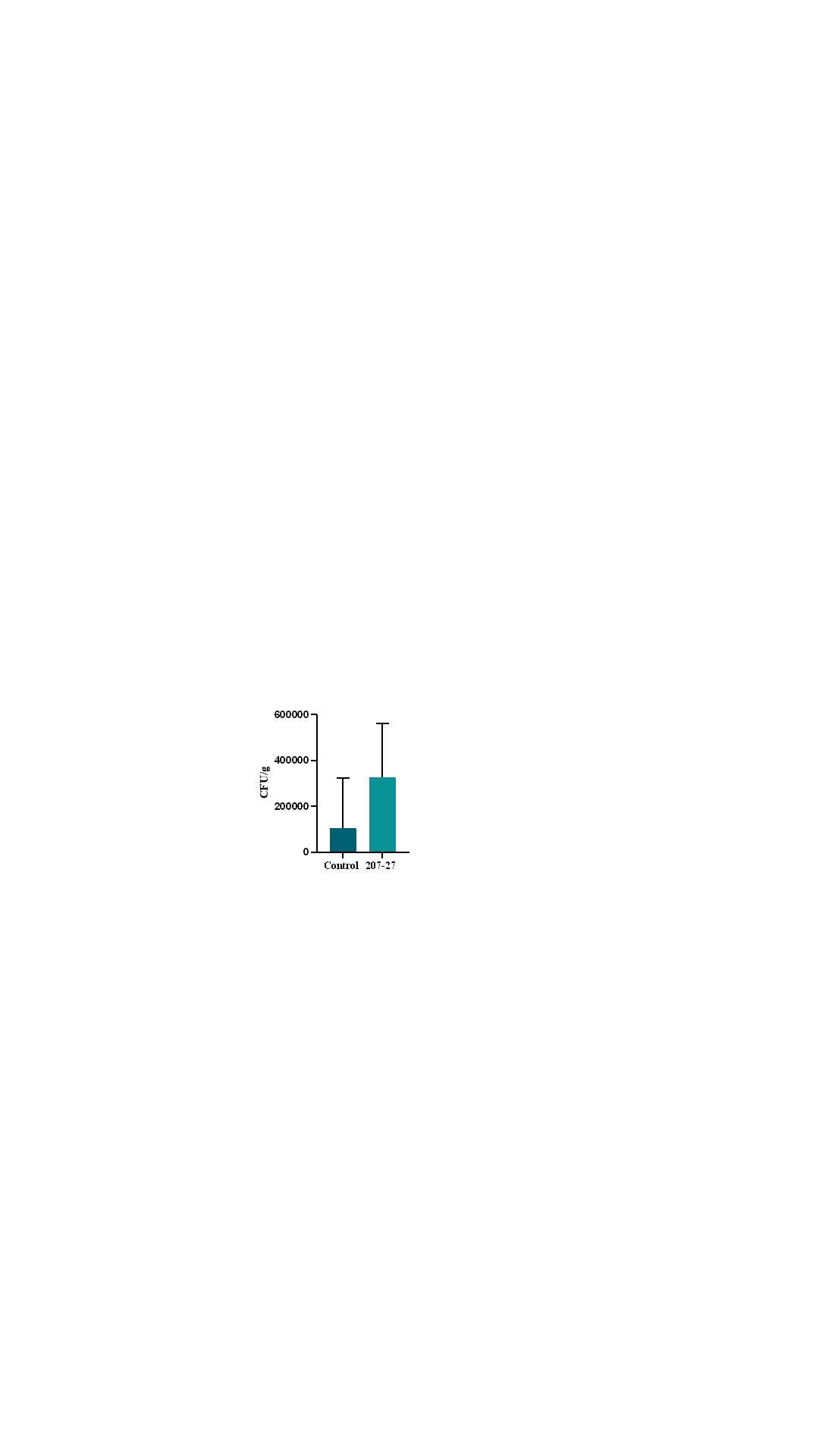

Supplement: Figure S5 — Effect of a 2-week oral gavage with Lacticaseibacillus paracasei 207-27 or normal saline on bacterial levels in the feces of 6-week-old BALB/c mice (n = 4–5). [file spectrum.02762-24-s0005.tiff]
