# Supplementary material for: Protective effect and mechanism of Lacticaseibacillus paracasei 207-27 administration on colitis in antibiotic-exposed mice in early life
Source: Microbiol Spectr. 2025 Oct 31;13(12):e02762-24. doi: 10.1128/spectrum.02762-24 (PMC12671169; doi:10.1128/spectrum.02762-24)
Supplement: Supplemental figures — Legends for Figures S1 to S5. [file spectrum.02762-24-s0007.docx]

**Supplementary Fig 1** Schematic of Grouping, Sample Size, and Intervention

**Supplementary Fig** **2** Local and systemic immunities after intervention on day 21. (*n*= 5–8) (A) Colonic IL-13 mRNA level. (B) Colonic IL-6 mRNA level. (C) Colonic IL-12(p40) mRNA level. (D) Colonic IL-23 mRNA level. (E) Colonic TNF-α mRNA level. (F) Colonic IFN-γ mRNA level. (G) Splenic IL-12(p40) mRNA level. (H) Serum IL-10 level (pg/mL). (I) Serum IL-13 level (pg/mL). (J) Serum TGF-β level (pg/mL). (K) Serum IL-23 level (pg/mL). **P*< 0.05 as conducted

**Supplementary Fig 3** Effects on metabolites on days 21 and 46. (*n*= 5) (A–C) SCFA concentrations on day 21 (propionic, butyric, and isovaleric acids). (D–L) SCFA concentrations on day 46 (acetic, propionic, butyric, isobutyric, valeric, isovaleric, and hexanoic acids). **P*< 0.05, ***P*< 0.01, ****P*< 0.001, *****P*< 0.0001 as conducted

**Supplementary Fig** **4** Local and systemic immunities after intervention on day 46. (*n*= 5–8) (A) Colonic IL-13 mRNA level. (B) Colonic IL-5 mRNA level. (C) Colonic IL-6 mRNA level. (D) Colonic IL-12(p40) mRNA level. (E) Splenic IL-10 mRNA level. (F) Splenic IL-13 mRNA level. (G) Splenic IL-12(p40) mRNA level. (H) Splenic IFN-γ mRNA level. (I) Serum IL-10 level (pg/mL). (J) Serum IL-13 level (pg/mL). (K) Serum IL-23 level (pg/mL). **P*< 0.05, ***P*< 0.01, ****P*< 0.001, *****P*< 0.0001 as conducted

**Supplementary Fig 5** Effect of a 2-week oral gavage with *Lacticaseibacillus paracasei* 207-27 or normal saline on bacterial levels in the feces of 6-week-old BALB/c mice (n=4-5).
